# Overexpression of C-type Natriuretic Peptide in Endothelial Cells Protects against Insulin Resistance and Inflammation during Diet-induced Obesity

**DOI:** 10.1038/s41598-017-10240-1

**Published:** 2017-08-29

**Authors:** Cho-Rong Bae, Jun Hino, Hiroshi Hosoda, Yuji Arai, Cheol Son, Hisashi Makino, Takeshi Tokudome, Tsutomu Tomita, Toru Kimura, Takashi Nojiri, Kiminori Hosoda, Mikiya Miyazato, Kenji Kangawa

**Affiliations:** 1 Department of Biochemistry, National Cerebral and Cardiovascular Center Research Institute, Suita, Osaka Japan; 2 Department of Regenerative Medicine and Tissue Engineering, National Cerebral and Cardiovascular Center Research Institute, Suita, Osaka Japan; 3 Department of Bioscience and Genetics, National Cerebral and Cardiovascular Center Research Institute, Suita, Osaka Japan; 4 Division of Endocrinology and Metabolism, National Cerebral and Cardiovascular Center, Suita, Osaka Japan; 5 Omics Research Center, National Cerebral and Cardiovascular Center, Suita, Osaka Japan; 6 Biobank, National Cerebral and Cardiovascular Center, Suita, Osaka Japan

## Abstract

The endogenous peptide C-type natriuretic peptide (CNP) binds its receptor, guanylyl cyclase B (GCB), and is expressed by endothelial cells in diverse tissues. Because the endothelial cells of visceral adipose tissue have recently been reported to play a role in lipid metabolism and inflammation, we investigated the effects of CNP on features of obesity by using transgenic (Tg) mice in which CNP was placed under the control of the Tie2 promoter and was thus overexpressed in endothelial cells (E-CNP). Here we show that increased brown adipose tissue thermogenesis in E-CNP Tg mice increased energy expenditure, decreased mesenteric white adipose tissue (MesWAT) fat weight and adipocyte hypertrophy, and prevented the development of fatty liver. Furthermore, CNP overexpression improved glucose tolerance, decreased insulin resistance, and inhibited macrophage infiltration in MesWAT, thus suppressing pro-inflammation during high-fat diet–induced obesity. Our findings indicate an important role for the CNP produced by the endothelial cells in the regulation of MesWAT hypertrophy, insulin resistance, and inflammation during high-fat diet–induced obesity.

## Introduction

Obesity has increased worldwide and is associated with diseases including dyslipidaemia, diabetes, cardiovascular disease, and various cancers^1^. Although adipose tissue is a major depot for the storage of fat in the form of triglycerides, its excess production disrupts the energy balance, leading to energy overconsumption. Adipose tissue contains mature adipocytes, pre-adipocytes, fibroblasts, endothelial cells, and a variety of immune cells, including macrophages^2^. In particular, by regulating the exchange of nutrients and oxygen between blood and tissues, endothelial cells are a key determinant of the growth and function of adipose tissue^3, 4^. Furthermore, inhibiting endothelial cell inflammation in visceral adipose tissue prevents alterations in adipocytes that contribute to obesity comorbidities^5^. Therefore, endothelial cells may affect adipose tissue during obesity-associated metabolic dysfunction.

Natriuretic peptides (NPs), including atrial NP (ANP), brain NP (BNP), and C-type NP (CNP), have attracted great attention as indicators of the metabolic regulation in the cardiovascular and renal systems^6–8^. CNP acts as a local factor by binding its specific receptor, guanylyl cyclase B (GCB), and is highly expressed by endothelial cells in the central nervous system and peripheral tissues^9–12^. CNP in the central nervous system reportedly regulates food intake and fat weight^13^. In addition, CNP has been shown to have anti-inflammatory, anti-mitogenic, and anti-fibrotic functions and to regulate vascular function^14–16^. NPs affect the homeostasis of glucose and lipid metabolism, partly through the reduction of adipogenesis^17^. Recently CNP has been implicated in regulation of lipogenesis in adipocytes^18^. We therefore hypothesized that endothelial cell–specific CNP overexpression may protect against the development of the obesity condition.

To this end, we generated transgenic (Tg) mice in which CNP was overexpressed under the control of the endothelial cell–specific Tie2 promoter^19^ (E-CNP) and used these mice to study the effects of CNP during high-fat diet (HFD)-induced and genetic obesity. We found that CNP overexpression increased energy expenditure, reduced mesenteric adipose tissue (MesWAT) hypertrophy, and improved insulin sensitivity and hepatic lipid metabolism during HFD-induced obesity. Furthermore, CNP overexpression had anti-inflammatory effects in the MesWAT of our mouse models of HFD-induced and genetic obesity.

## Results

### Endogenous *CNP* and *GCB* expression increased in the peripheral tissues of HFD-fed mice compared with STD-fed mice

**Figure 1:**
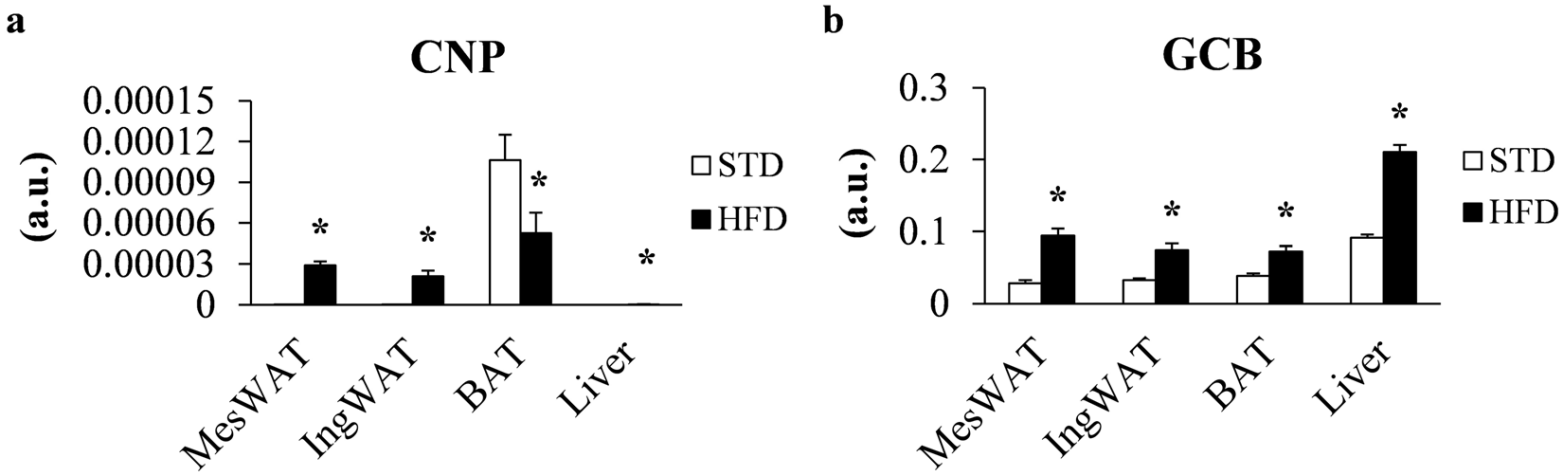
*CNP* and *GCB* mRNA expression in peripheral tissues of standard diet (STD) and high-fat diet (HFD)-fed mice. (**a**) *CNP* and (**b**) *GCB* mRNA expression in the mesenteric (MesWAT), and inguinal (IngWAT) white adipose tissue, brown adipose tissue (BAT), and liver in STD-fed and HFD-fed mice. a.u., arbitrary units (copy number of gene of interest/copy number of reference gene [ribosomal protein 36B4]). *n* = 10 (**a** and **b**). *P < 0.05.

In the HFD-fed mice, *CNP* mRNA expression significantly increased, as in peripheral tissues, in mesenteric white adipose tissue (MesWAT), inguinal white adipose tissue (IngWAT), and liver compared with standard diet (STD)-fed mice, whereas in brown adipose tissue (BAT) *CNP* mRNA expression was decreased compared with STD-fed mice (Fig. 1a). In addition, *GCB* mRNA expression in the MesWAT, IngWAT, BAT, and liver of HFD-fed mice was significantly greater compared with that in STD-fed mice (Fig. 1b). These results indicate that endogenous *CNP* and *GCB* mRNA expression differ in the peripheral tissues of HFD-fed mice compared with STD-fed mice, suggesting that CNP expression affects obesity. Therefore, we examined the role of endothelial cell–specific overexpression of CNP in HFD-induced obesity.

### *CNP* mRNA expression is upregulated in the tissues of E-CNP Tg mice

**Figure 2:**
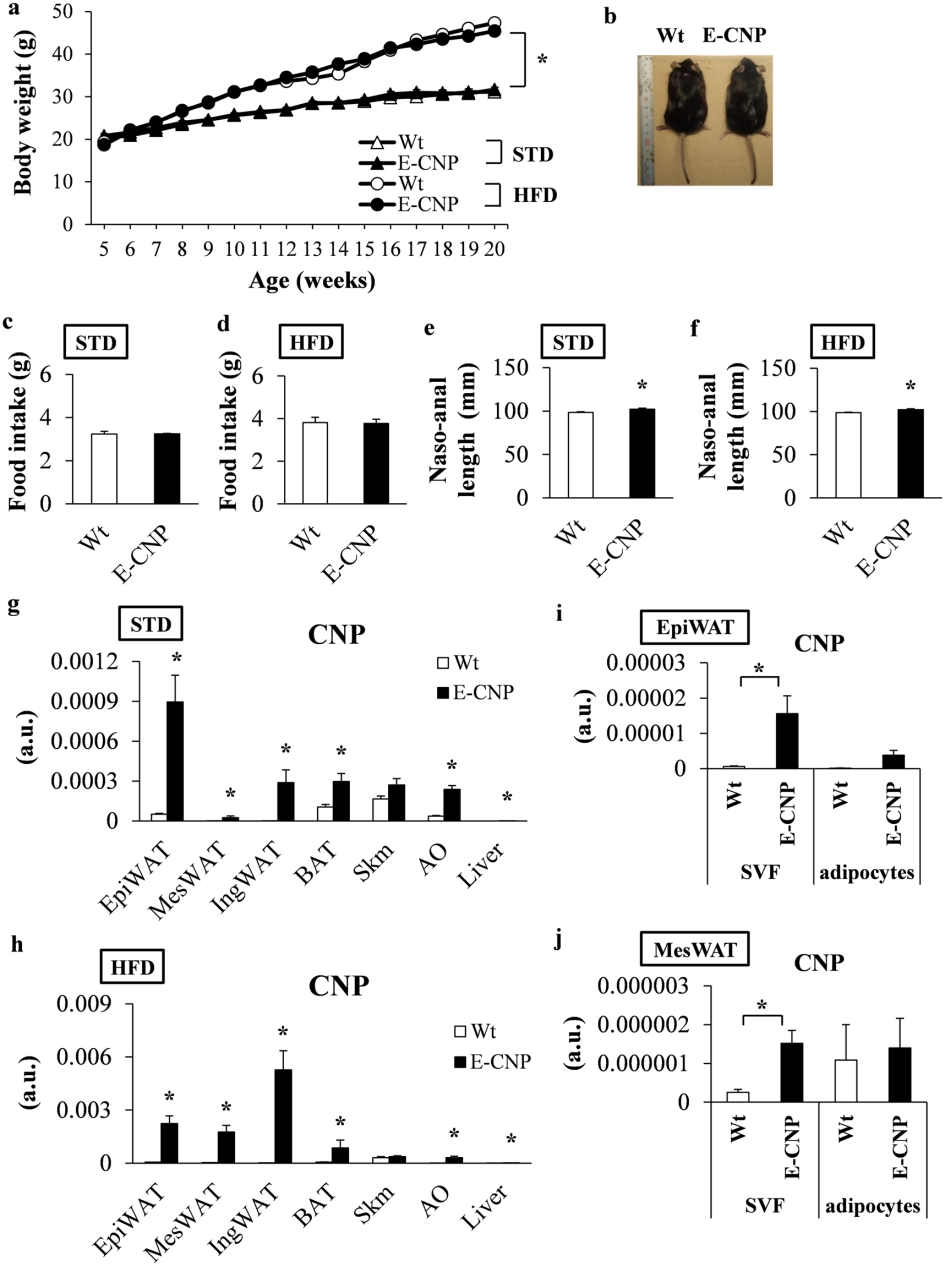
Characterization of wild-type (Wt) mice and transgenic mice that overexpress C-type natriuretic peptide specifically in their endothelial cells (E-CNP Tg mice) fed a STD or HFD. (**a**) Body weight over time. (**b**) Macroscopic appearance, (**c** and **d**) food intake, and (**e** and **f**) naso-anal length of Wt and E-CNP Tg mice (age, 20 weeks) fed STD and HFD. (**g** and **h**) *CNP* mRNA expression in the epididymal white adipose tissue (EpiWAT), MesWAT, IngWAT, BAT, skeletal muscle (Skm), aorta (AO) and liver from Wt and E-CNP Tg (**g**) STD-fed and (**h**) HFD-fed mice (age, 20 weeks) was measured by using quantitative real-time polymerase chain reaction (qPCR) analysis. (**i** and **j**) *CNP* mRNA expression in the stromal vascular fraction (SVF) and mature adipocytes of the (**i**) EpiWAT and (**j**) MesWAT of HFD-fed mice. a.u., arbitrary units (copy number of gene of interest/copy number of reference gene [ribosomal protein 36B4]). *n* = 10 (**a**,**c**–**h**); *n* = 6 (**i** and **j**). *P < 0.05 between STD- and HFD-fed mice (**a**) or between Wt and E-CNP Tg mice (**e**–**j**).

The body weights of both wild-type (Wt) and E-CNP Tg mice on HFD were increased compared with their STD-fed counterparts but did not differ between Wt and E-CNP Tg mice on the same diet from 5 to 20 weeks of age (Fig. 2a and b). Food intake remained similar among all groups regardless of diet (Fig. 2c and d). However, the naso-anal length was greater in E-CNP Tg than Wt mice on both STD and HFD (Fig. 2e and f), perhaps reflecting the positive effect of CNP on bone growth^20^.

We then examined *CNP* mRNA expression in various tissues and found that it was increased in the epididymal white adipose tissue (EpiWAT), MesWAT, IngWAT, BAT, aorta (AO), and liver of 20-week-old STD-fed and HFD-fed E-CNP Tg mice compared with their Wt controls (Fig. 2g and h). However, *GCB* mRNA expression did not differ between groups or tissues (Supplementary Figure S1a and b).

Visceral adipose tissue demonstrates characteristic changes in quantity and features during obesity; adipose tissue is composed of mature adipocytes and nonadipocyte cells; these cells constitute the stromal vascular fraction (SVF)^21^, which is rich in pre-adipocytes, endothelial cells, macrophages, and diverse other cells. We isolated the SVF and mature adipocytes from EpiWAT and MesWAT (i.e., visceral fat depots) of HFD-fed mice and measured the *CNP* and *GCB* mRNA expression in these cell populations. CNP expression in mature adipocytes was similar between groups but was significantly greater in the SVF of EpiWAT (Fig. 2i) and MesWAT (Fig. 2j) from E-CNP Tg mice than in those from Wt mice. In comparison, *GCB* mRNA expression in the EpiWAT and MesWAT remained similar between SVF and mature adipocytes and between groups (Supplementary Figure S1c and d).

### E-CNP Tg mice demonstrate increased energy expenditure due to BAT thermogenesis during HFD-induced obesity

**Figure 3:**
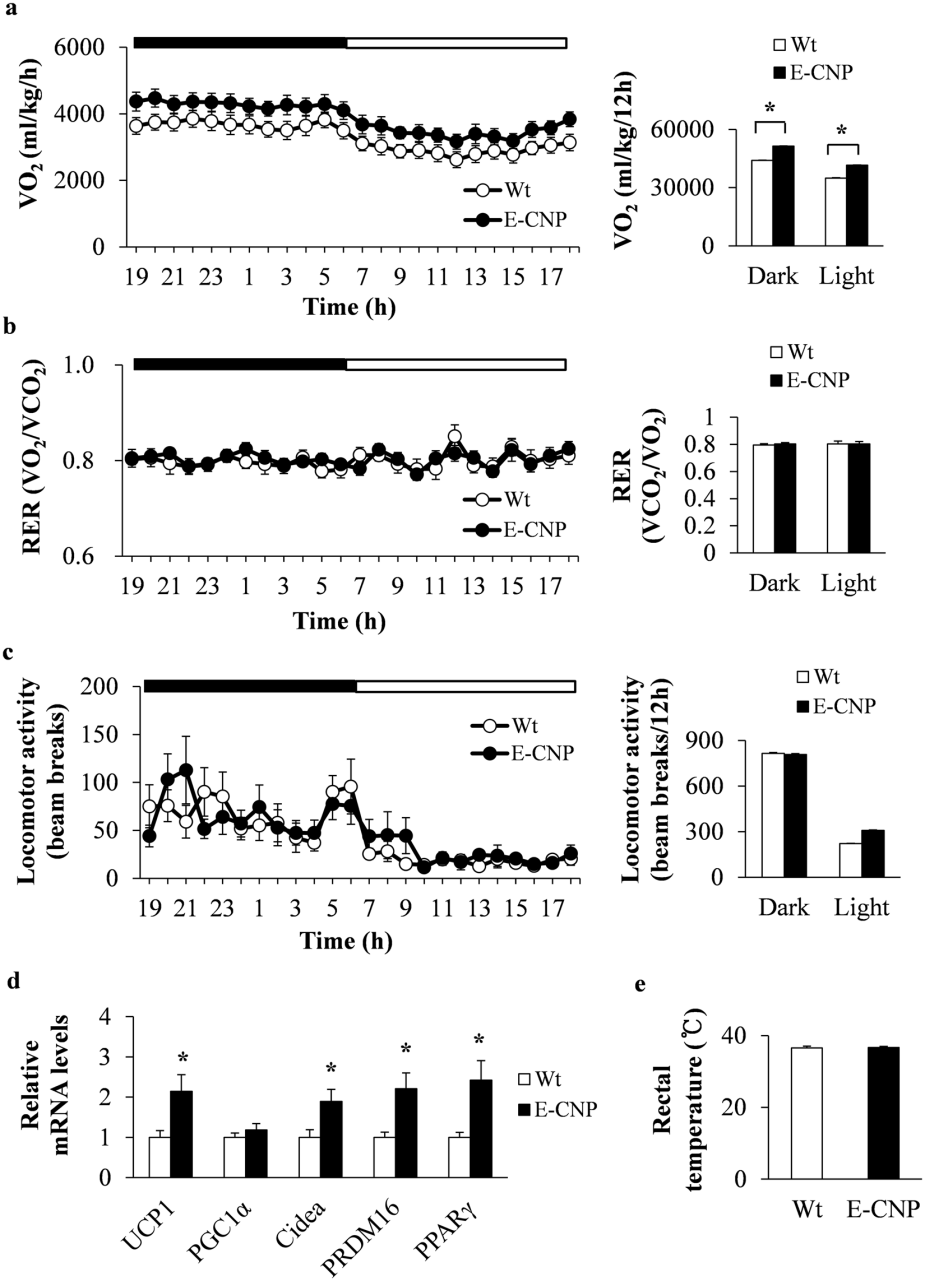
Energy metabolism of HFD-fed E-CNP Tg mice. (**a**) Oxygen consumption (VO_2_), (**b**) Respiratory exchange ratio (RER), and (**c**) locomotor activity in Wt and E-CNP Tg HFD-fed mice (age, 20 weeks). (**d**) qPCR analysis of the expression of thermogenesis-related genes in BAT. (**e**) Rectal temperature of Wt and E-CNP Tg mice. *n* = 10 (**a**–**c**); *n* = 10–12 (**d**); *n* = 5 (**e**). *P < 0.05.

We then investigated the metabolic effects of CNP overexpression in STD- and HFD-fed E-CNP Tg mice. Oxygen consumption (VO_2_), the respiratory exchange ratio (RER), and locomotor activity did not differ between groups of STD-fed mice (Supplementary Figure S2a–c). However, VO_2_ was increased during both the light and dark phases in HFD-fed E-CNP Tg mice compared with Wt mice, whereas RER and locomotor activity were similar between groups (Fig. 3a–c). Because BAT has recently been recognized as an important player in energy metabolism^22^, we analysed the expression of various thermogenesis-related genes in the BAT of Wt and E-CNP Tg mice. Compared with Wt mice, E-CNP Tg mice showed increased mRNA levels of uncoupling protein 1 (*UCP1*), cell death–inducing DNA fragmentation factor α-like effector A (*Cidea*), positive regulatory domain–containing protein 16 (*PRDM16*), and peroxisome proliferator–activated receptor γ (*PPARγ*) (Fig. 3d). In addition, the inguinal WAT of rodents contains brown-like adipocytes^23^, in which excess energy is dissipated directly by UCP1-associated uncoupling of fatty-acid oxidation from ATP production^24^. We therefore evaluated the mRNA level of *UCP1*, a transcriptional regulator of BAT development, in the IngWAT of our mice but found that it did not differ between groups (Supplementary Figure S3). In addition, rectal temperature was similar between groups (Fig. 3e). These data suggest that the increased thermogenic activity in their BAT led to elevated energy expenditure in E-CNP Tg mice.

### Adipocyte hypertrophy and lipogenesis in MesWAT are suppressed during HFD-induced obesity in E-CNP Tg mice

**Figure 4:**
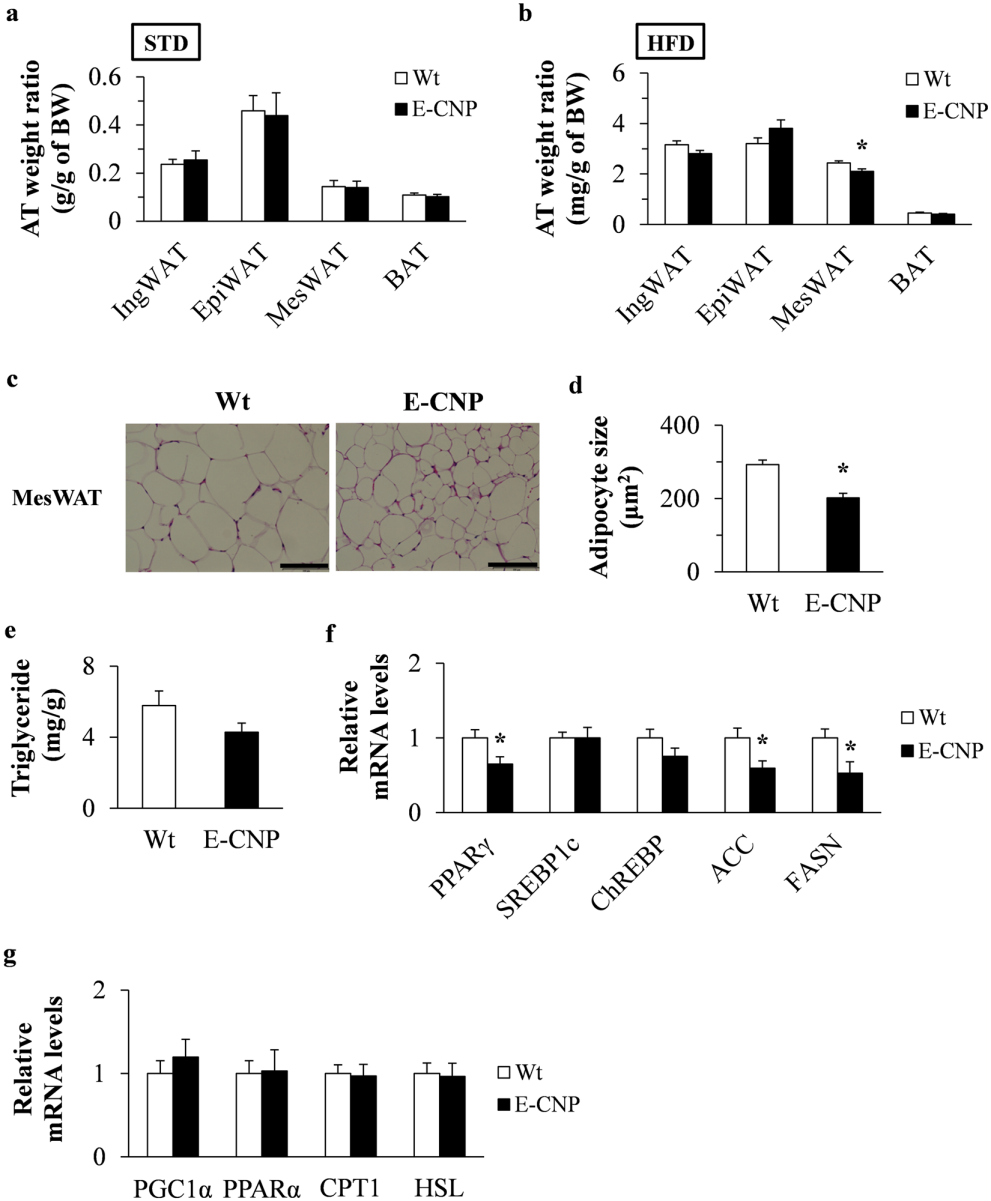
MesWAT weight and lipogenesis in HFD-fed E-CNP Tg mice. (**a** and **b**) Adipose tissue weight normalized to body weight in (**a**) STD-fed and (**b**) HFD-fed mice. (**c** and **d**) Size of adipocytes in MesWAT. Scale bar, 100 μm. (**e**) Triglyceride content of MesWAT. (**f** and **g**) qPCR analysis of mRNA levels in MesWAT of genes involved in (**f**) lipogenesis and (**g**) fatty acid β-oxidation and lipolysis. *n* = 10 (**a,** **b,** **d–** **g**); *n* = 4 (**c**). *P < 0.05.

The ratio between adipose tissue weight and body weight was similar between Wt and Tg STD-fed mice (Fig. 4a), whereas MesWAT–body weight ratio and adipocyte size both were significantly decreased in HFD-fed E-CNP Tg mice compared with Wt mice (Fig. 4b–d). In addition, triglyceride content tended to be decreased in E-CNP Tg mice (Fig. 4e), and the expression of lipogenesis-related genes including *PPARγ*, acetyl-CoA carboxylase (*ACC*), and fatty acid synthase (*FASN*) and the FASN protein level all were decreased in the MesWAT of E-CNP Tg mice compared with their Wt counterparts (Fig. 4f and Supplementary Figure S4). In contrast, fatty acid β–oxidation related genes such as peroxisome proliferator–activated receptor γ coactivator 1α (*PGC1α*), peroxisome proliferator–activated receptor α (*PPARα*), carnitine palmitoyltransferase 1 (*CPT1*), and hormone-sensitive lipase (*HSL*) showed similar expression levels between groups (Fig. 4g). Furthermore, E-CNP Tg mice had decreased serum levels of triglycerides, total cholesterol, insulin, and leptin and an increased adiponectin level, compared with Wt mice (Supplementary Table S1). Together these findings suggest that the overexpression of CNP in endothelial cells reduced the increase in the MesWAT–body weight ratio during HFD-induced obesity accompanying both reducing adipocyte size and modulating adipokines.

### E-CNP Tg mice have improved glucose tolerance and decreased insulin resistance during HFD-induced obesity

**Figure 5:**
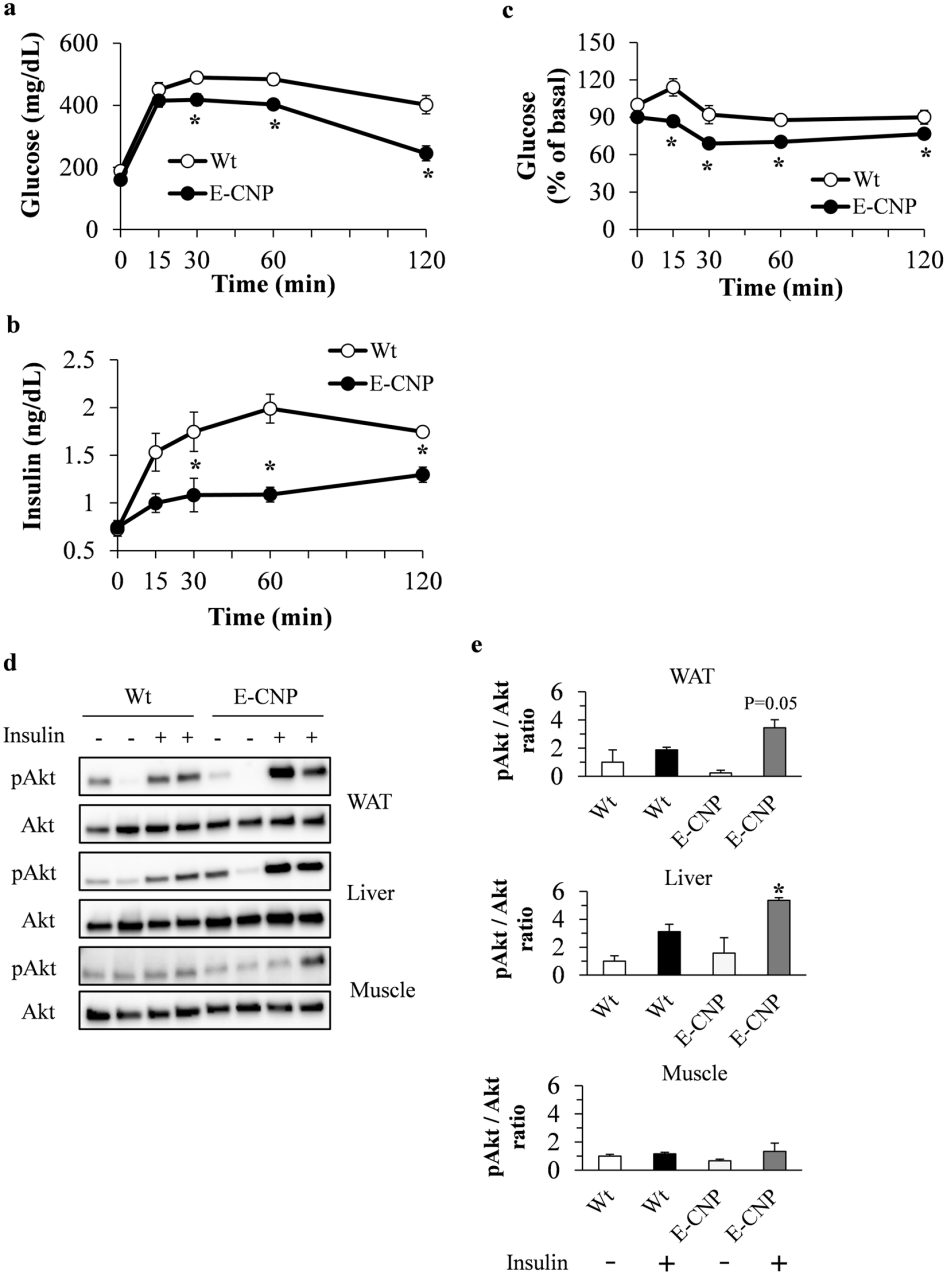
Insulin sensitivity in HFD-fed E-CNP Tg mice. (**a**) Results of glucose tolerance test (GTT), (**b**) serum insulin levels during GTT, and (**c**) results of insulin tolerance test (ITT) in HFD-fed mice (age, 20 weeks). (**d** and **e**) Activation of Akt phosphorylation in WAT, liver, and muscle after insulin injection (1 U/kg IP, 8 min). (**d**) Western blot analysis of phosphorylated (Ser^473^) Akt (pAkt) levels under control (PBS [-]) and insulin-treated ( + ) conditions in WAT, liver, and muscle extracts. (**e**) Densitometric quantitation of the pAkt:Akt ratio in WAT, liver, and muscle extracts. The gels were run under the same experimental conditions and were cropped to show protein bands corresponding to pAkt, Akt, and tubulin as indicated. *n* = 12–14 (**a** and **c**); *n* = 7 (**b**); *n* = 4 (**d** and **e**). *P < 0.05 between Wt and E-CNP mice (**a**–**c**) or compared with value for Wt mice without insulin injection (**e**).

We assessed glucose tolerance and insulin resistance in E-CNP Tg and Wt mice fed STD or HFD. In STD-fed mice, glucose levels during GTT and ITT did not differ between groups (Supplementary Figure S5). However, in HFD-fed mice, glucose and insulin levels during GTT were lower in E-CNP Tg mice than in Wt mice (Fig. 5a and b), as was the glucose level during ITT (Fig. 5c). We found that insulin-stimulated protein kinase B (Akt) phosphorylation was significantly increased in liver from insulin-stimulated E-CNP Tg compared with Wt mice (Fig. 5d and e). In addition, immunohistochemical staining revealed that the insulin- and Ki67-positive areas in pancreatic islets were significantly smaller in E-CNP Tg mice than in Wt mice (Supplementary Figure S6). These results indicate that E-CNP Tg mice with HFD-induced obesity have improved glucose tolerance and decreased insulin resistance.

### Inflammation during HFD-induced obesity is ameliorated in E-CNP Tg mice

**Figure 6:**
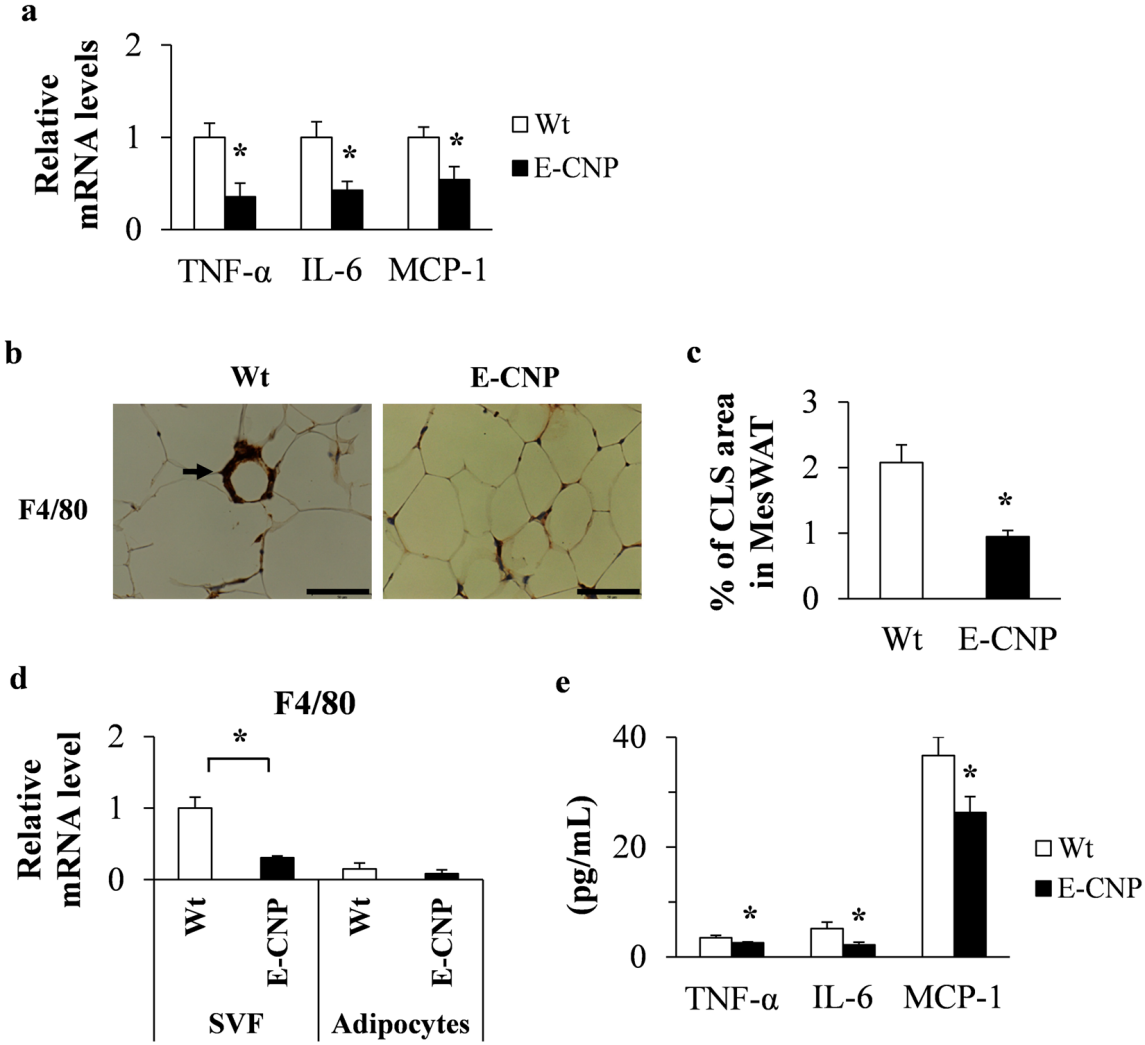
Inflammation in HFD-fed E-CNP Tg mice. (**a**) Expression of inflammatory cytokines in MesWAT was determined by qPCR analysis and normalized against the mRNA level of the ribosomal protein 36B4. (**b** and **c**) Immunohistochemical analysis of crown-like structures (CLSs) in E-CNP Tg mice by using anti-F4/80 staining. Scale bar, 50 μm. (**d**) Expression of inflammatory cytokines in mature adipocytes and the stromal vascular fraction (SVF) of MesWAT as determined by qPCR analysis. (**e**) Serum levels of inflammatory cytokines were measured by ELISA. *n* = 10 (**a**); *n* = 3 (**b** and **c**); *n* = 4 (**d**); *n* = 10 (**e**). *P < 0.05.

We then investigated the anti-inflammatory effects of CNP overexpression in the endothelial cells. Compared with that in Wt mice, the MesWAT of E-CNP Tg mice had significantly lower mRNA levels of tumour necrosis factor α (*TNF-α*), interleukin 6 (*IL-6*), and monocyte chemoattractant protein 1 (*MCP-1*) (Fig. 6a). Crown-like structures arise when F4/80-positive macrophages infiltrate adipose tissue and surround adipocytes^25^. Fewer F4/80-positive macrophages infiltrated into the MesWAT (Fig. 6b and c) and *F4/80* mRNA levels in the SVF of MesWAT (Fig. 6d) were lower in E-CNP Tg mice than in Wt mice. Furthermore, serum concentrations of inflammatory cytokines, including TNF-α, IL-6, and MCP-1, were significantly lower in E-CNP Tg mice (Fig. 6e) and may be responsible for the anti-inflammatory effects in these mice.

### Hepatic features of E-CNP Tg mice during HFD-induced obesity

**Figure 7:**
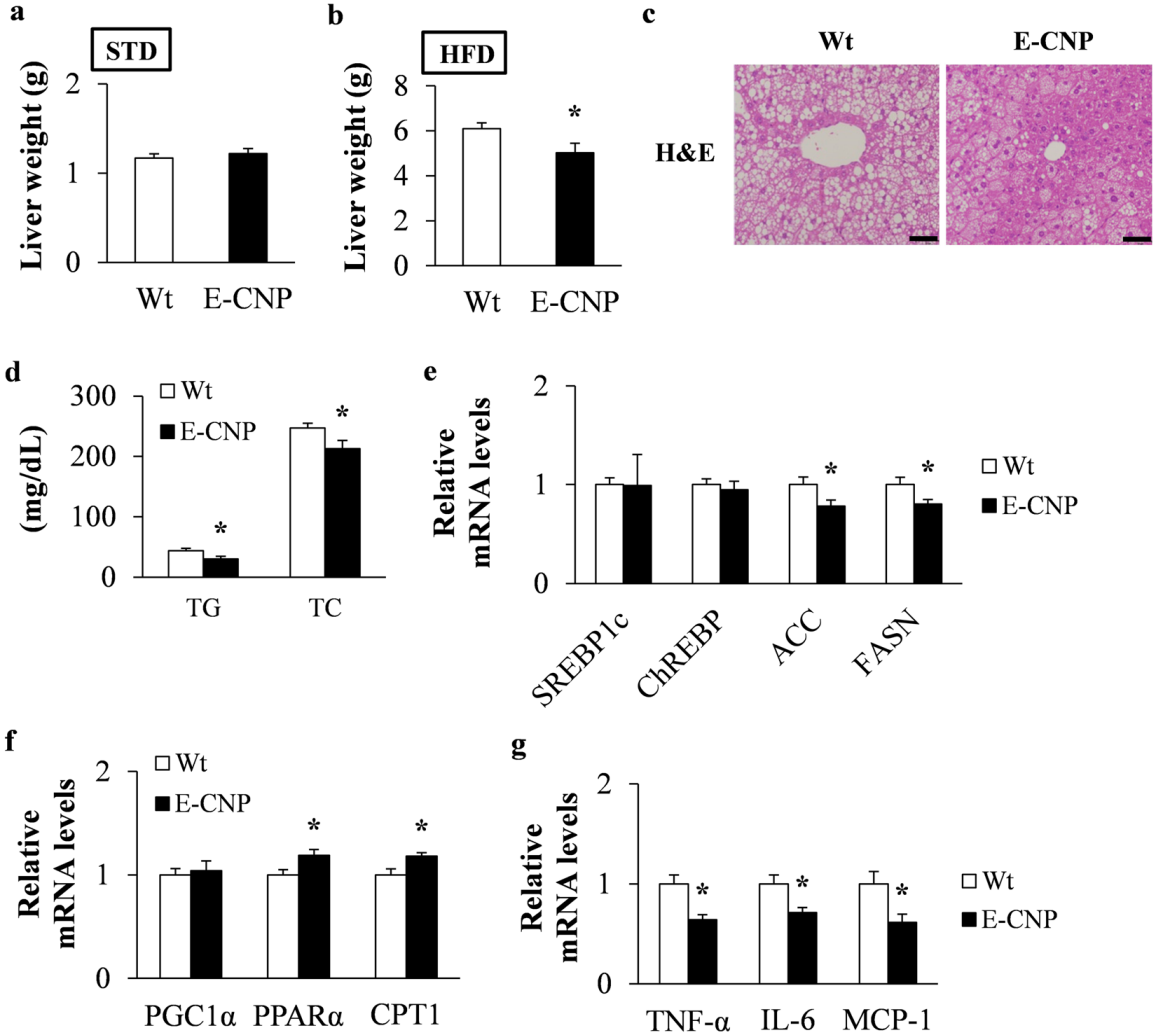
Characterization of liver in HFD-fed E-CNP Tg mice. (**a** and **b**) The liver weight of Wt and E-CNP Tg mice fed (**a**) STD and (**b**) HFD. (**c**) Liver sections stained with hematoxylin and eosin (H&E). Scale bar, 50 μm. (**d**) Triglyceride (TG) and total cholesterol (TC) content in liver. The expression of genes related to (**e**) lipogenesis and (**f**) fatty acid β-oxidation and of (**g**) pro-inflammatory genes in liver was measured by using qPCR analysis. *n* = 10 (**a** and **b**); *n* = 6 (**c**); *n* = 9 (**d**); *n* = 10 (**e**–**g**); *P < 0.05.

The liver is a highly vascular organ, receiving as much as 25% of the total cardiac output in animals at rest^26^, and is a key player in lipid metabolism^27^. Whereas liver weight did not differ between Wt and Tg STD-fed mice (Fig. 7a), liver weight as well as triglyceride and total cholesterol contents were significantly decreased in HFD-fed E-CNP Tg compared with Wt mice (Fig. 7b–d). These findings prompted us to investigate indicators of hepatic lipid metabolism and pro-inflammatory markers. E-CNP Tg mice showed significantly decreased expression of lipogenic enzymes such as *ACC* and *FASN* (Fig. 7e) but increased expression of the fatty acid β-oxidation related factors *PPARα* and *CPT1* (Fig. 7f). mRNA levels of the pro-inflammatory markers *TNF-α*, *IL-6*, and *MCP-1* were significantly decreased in the liver of E-CNP Tg compared with Wt mice (Fig. 7g). These combined results indicate that the inflammation typically associated with HFD-induced obesity was decreased in E-CNP Tg mice.

### Decreased inflammation in OBEC mice

**Figure 8:**
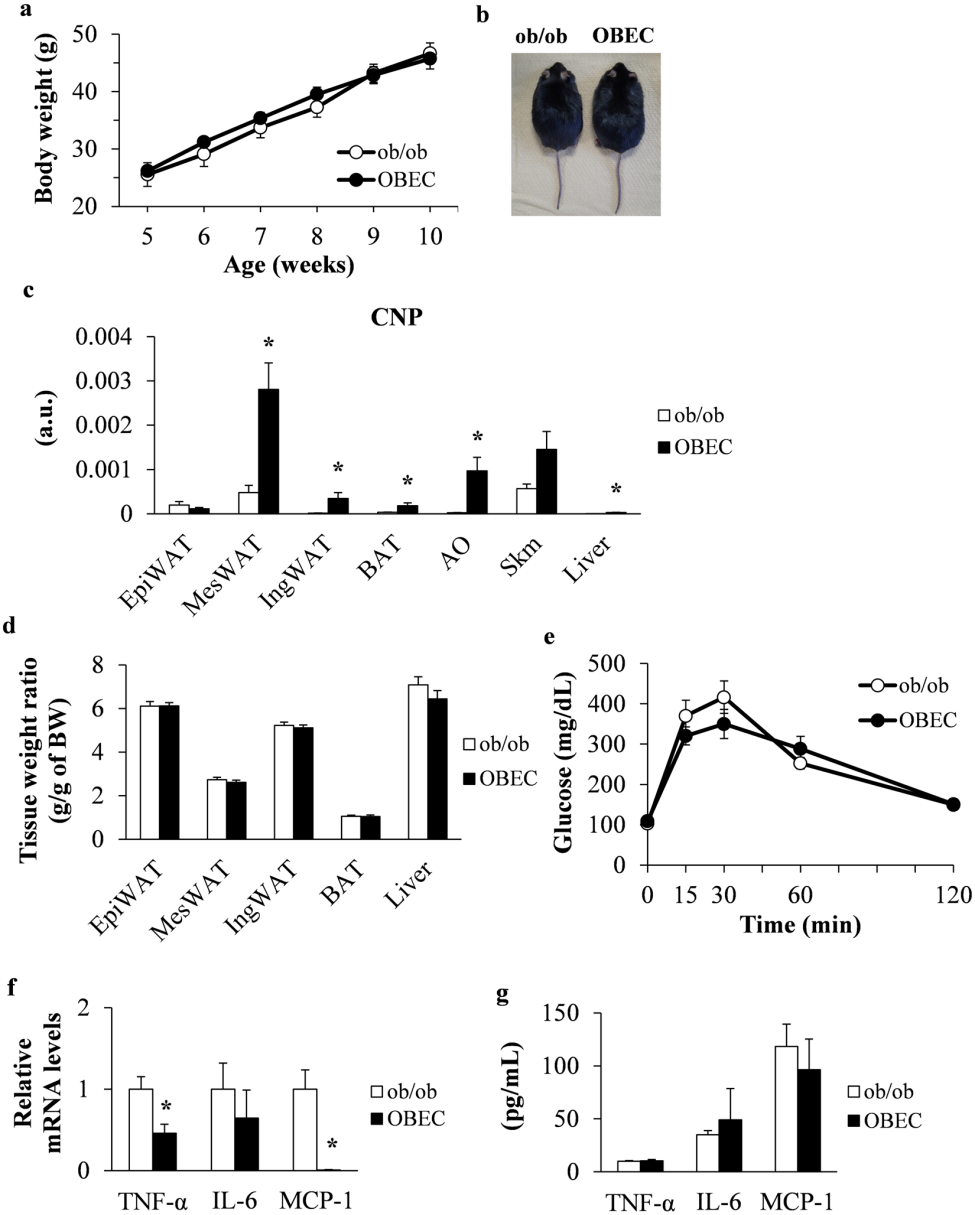
Characteristics of OBEC mice, including pro-inflammatory cytokine expression in MesWAT. (**a**) Body weight over time. (**b**) Macroscopic appearance and (**c**) *CNP* mRNA levels in tissues of ob/ob and OBEC mice at 10 weeks of age. (**d**) Tissue weight ratio and (**e**) glucose tolerance testing in Wt and OBEC mice. (**f**) Expression of pro-inflammatory cytokines in MesWAT was determined by qPCR analysis. (**g**) Serum levels of pro-inflammatory cytokines was measured by ELISA. a.u., arbitrary units (copy number of gene of interest/copy number of reference gene [ribosomal protein 36B4]). *n* = 10 (**a**,**c**–**g**). *P < 0.05.

We then sought to confirm the effects of endothelialcell–specific CNP overexpression in another obesity model. One of the most studied models in obesity research, ob/ob mice are genetically deficient in leptin^28^, which has a prominent role in the development of obesity^29^. In particular, ob/ob mice display hyperphagia, obesity, hyperglycaemia, and insulin resistance^30^. We crossed E-CNP heterozygous (ob/+ ∙ E-CNP Tg) mice with leptin homozygous (ob/ob) mice to generate OBEC mice, which are leptin-deficient and express CNP in endothelial cells. Body weight and macroscopic appearance were similar between ob/ob and OBEC mice fed STD (Fig. 8a and b). Compared with ob/ob mice, OBEC mice had significantly increased *CNP* mRNA expression in MesWAT, IngWAT, BAT, AO, and liver (Fig. 8c). However, *GCB* mRNA expression remained similar between groups and tissues (Supplementary Figure S7). In addition, the tissue–body weight ratio and glucose level during GTT were similar between these groups (Fig. 8d and e). However, whereas OBEC mice showed decreased expression of pro-inflammatory genes, such as *TNF-α* and *MCP-1*, in MesWAT (Fig. 8f), serum levels of these pro-inflammatory cytokines did not differ between groups (Fig. 8g). Therefore, CNP overexpression in endothelial cells decreased pro-inflammation in the MesWAT of genetically obese mice.

## Discussion

In this study, we examined the effects of CNP overexpression in the endothelial cells of mice with HFD-induced (E-CNP) or genetic (OBEC) obesity. Our results show that BAT thermogenesis was stimulated in E-CNP Tg mice, leading to increased energy expenditure, decreased MesWAT–body weight ratio and insulin resistance, and inhibition of fatty liver during HFD-induced obesity. In addition, the overexpression of CNP in endothelial cells ameliorated HFD-induced and genetic obesity-associated inflammation.

Food intake was similar between Wt and E-CNP Tg mice, but E-CNP Tg mice had higher energy expenditure, as indicated by their increased oxygen consumption. This increased energy expenditure was accompanied by increased expression of thermogenesis-related genes, including *UCP1*, *Cidea*, *PRDM16*, and *PPARγ*, in BAT. Increased energy expenditure typically is associated with a loss of adipose tissue weight^31^. Therefore, this decreased MesWAT–body weight ratio reflects the increased energy expenditure in E-CNP Tg mice.

Visceral adipose tissue might be an important clinical target for manipulating metabolism^32^. The lipogenic regulation of lipid metabolism modulates endothelial cell function associated with obesity^33^. Consistent with this finding, the MesWAT of E-CNP Tg mice demonstrated decreased adipocyte hypertrophy and lipogenesis. Adipose tissue and liver are key tissues in the context of lipid homeostasis^34^; accordingly, decreased adipocyte size was positively associated with decreased hepatic lipid content in our E-CNP Tg mice. Lipid accumulation in visceral adipose tissue and liver is crucial in the regulation of insulin sensitivity, thus suggesting a link between obesity-related insulin resistance and an increased risk of diabetes^35, 36^. Furthermore, decreased hepatic weight and insulin activity are both hallmarks of non-alcoholic fatty liver disease^37^. Therefore, CNP overexpression in endothelial cells may both decrease hepatic lipid accumulation and improve the response to insulin.

The E-CNP Tg mice were protected against not only HFD-induced obesity but also insulin resistance. The results of the GTT, ITT, and insulin-stimulated Akt phosphorylation assay were improved in E-CNP Tg compared with Wt mice. Given that adipocyte hypertrophy and the infiltration of macrophages into adipose tissue promote obesity-associated insulin resistance^38^, the decreased insulin resistance in E-CNP Tg mice may reflect not only decreased MesWAT accumulation but also decreased inflammation.

The vascular injury induced by high blood glucose levels can lead to endothelial cell dysfunction and the production of inflammatory cytokines^39, 40^. Therefore, we confirmed that the anti-inflammatory effects of endothelialcell–specific CNP overexpression were present during both HFD-induced and genetic obesity. However, the degree of anti-inflammatory effect differed between E-CNP Tg and OBEC mice: whereas OBEC mice showed decreased pro-inflammatory mRNA levels in MesWAT only, E-CNP Tg mice had increased anti-inflammatory effects in tissues as well as serum. The endothelial cells and macrophages in adipose tissue play critical roles in inflammatory responses^41^, and endothelial cells from visceral adipose tissue are both active participants in and regulators of inflammation^42, 43^. In addition, important features of an effective inflammatory response include increased macrophage infiltration and concomitantly increased TNF-α and IL-6 expression^44^; in addition, the pro-inflammatory compound MCP-1 is important for recruiting macrophages into adipose tissue^45^. However, the mechanism underlying the different in anti-inflammatory effects between E-CNP Tg and OBEC mice is unclear currently but may reflect their differing levels of obesity. Regardless of the type of obesity involved, we surmise that endothelial cell–specific overexpression of CNP reduces inflammation.

Interestingly, our results revealed beneficial effects of endothelial cell–specific overexpression of CNP in the liver of mice with HFD-induced obesity. Previous studies showed that both ANP and BNP can decrease adipocyte hypertrophy, inflammation, and insulin resistance and exert hepatoprotective effects^46^. MesWAT is drained by the portal vein, whereas the blood from all other abdominal fat, including perigonadal, bypasses the liver^47^. The portal drainage of cytokines, adipokines, and free fatty acids released from visceral adipose tissue directly to the liver may significantly influence metabolism^48^. Therefore, the vasculature is a primary site at which inflammation occurs in obesity, and HFD-elicited vascular inflammation contributes to the development of obesity-associated metabolic disorders^49^. Furthermore, recent studies have recognized that characteristics and functions of the sheath of adipose tissue that surrounds blood vessels (perivascular adipose tissue, PVAT)^50^ differ according to its specific location^51^. PVAT regulates endothelial function^52^, and appears to be altered in obesity and diabetes mellitus, during which the PVAT expands, accumulates inflammatory cells, and alters the production of various adipokines and inflammatory cytokines^53, 54^. Therefore, endothelial cell–specific CNP overexpression, which was increased in highly vascularized tissues such as BAT^55^, MesWAT^56^, and liver of our Tg mice, may protect against metabolic disorders in HFD-induced obesity.

Lastly, the NP family members ANP and BNP contribute to the homeostatic maintenance of blood pressure^57, 58^. In comparison, the activity of exogenous CNP is weaker than that of ANP, and exogenous CNP has activities without effects on blood pressure^59–61^; both of these possibilities are consistent with the results seen in our endothelial cell–specific CNP Tg mice (Supplementary Table S2). In addition, deletion of endothelium-derived CNP recently was shown to increase blood pressure^62, 63^. Therefore, CNP’s effects on the cardiovascular system may differ depending on its source (that is, exogenous or endogenous). To confirm this hypothesis, our futures studies will focus on the precise function of CNP in the regulation of the cardiovascular system.

In conclusion, the overexpression CNP in endothelial cells promoted BAT thermogenesis, leading to increased energy expenditure and decreased MesWAT adipocyte hypertrophy, fatty liver, insulin resistance, and inflammation in HFD-fed Tg mice.

## Materials and Methods

### Animals

C57BL/6 J mice were obtained from CLEA Japan (Tokyo, Japan). Ob/ob mice (B6.V-Lep^ob^/J) were obtained from the Jackson Laboratory (stock 000632; Bar Harbor, ME, USA). All experiments involving animals were approved in advance by the Animal Care and Use Committee of the National Cerebral and Cardiovascular Center Research Institute (Osaka, Japan) and were performed in accordance with approved guidelines. The laboratory animal facilities of the National Cerebral and Cardiovascular Center Research Institute comply with the “Basic Policies for the Conduct of Animal Experimentation” of the Ministry of Health, Labour, and Welfare, as assessed by the Center for Accreditation of Laboratory Animal Care and Use, Japan Health Sciences Foundation. All mice were housed under a 12:12-h light:dark cycle and had unrestricted access to STD (12 kcal% fat, 29 kcal% protein, and 59 kcal% carbohydrate; CE2, CLEA Japan) or HFD (57 kcal% fat, 20 kcal% protein, and 23 kcal% carbohydrate; High-Fat Diet 32, CLEA Japan). For the diet-induced obesity model, Wt and E-CNP Tg mice were fed HFD beginning at 5 weeks of age; Ob/ob and OBEC mice were fed STD until 10 weeks of age. All mice had unrestricted access to feed and water and were weighed once weekly. Body length was measured as the nose-to-anus length at necropsy, and MesWAT was stripped from the duodenum to the colon. When stated, mice were fasted for 16 h before being euthanized.

### Generation of E-CNP and OBEC mice

The E-CNP Tg construct was generated through conventional methods; the coding region of human CNP cDNA (clone RBd49C10; RIKEN BioResource Center, Tsukuba, Japan) was inserted into a plasmid vector (pBluescript) to place its expression under the control of the Tie2 promoter–enhancer (UNITECH, Chiba, Japan). The resulting E-CNP Tg construct was purified and microinjected into the pronucleus of C57BL/6 J mouse embryos by using standard techniques. E-CNP Tg F1 mice were identified by using PCR analysis and then mated with C57BL/6 J mice to expand the population of E-CNP Tg mice. All experiments that used E-CNP Tg mice were performed on male Tg mice and their male Wt littermates.

To generate OBEC mice, two *in vitro* fertilization (IVF) steps were performed by using standard techniques. The first IVF step involved ob/ob and E-CNP Tg mice to create ob/+∙ E-CNP Tg mice. In the second IVF step, ob/+∙ E-CNP Tg and ob/ob mice were used to produce ob/ob ∙ E-CNP Tg (OBEC) mice. Experiments involving OBEC mice used male OBEC mice and their male ob/ob littermates from the second IVF step.

### Isolation of SVF and mature adipocytes

MesWAT was fractionated as described previously^64^, with slight modification. Briefly, fat pads were isolated from 20-week-old HFD-fed Wt and E-CNP Tg mice; a maximum of 1 g of tissue was digested with 20 mg of collagenase type VIII (Sigma, St Louis, MO, USA) in Krebs–Ringer bicarbonate HEPES buffer containing 1% bovine serum albumin (Sigma) at 37 °C for 1 h. After centrifugation, the upper layer was harvested as the source of mature adipocytes, and the SVF was obtained as the precipitated cells.

### Metabolic assessment

Using a metabolic monitoring system (CLAMS; Columbus Instruments, Columbus, OH, USA), we assessed the VO_2_, RER, and locomotor activity of 20-week-old STD- or HFD-fed Wt and E-CNP Tg mice for 1 week. Body temperature was measured by inserting a sensor (measurement range, 22–42 °C; measurement error, ±0.1 °C) into the rectum (ATB-1100, Nihon Kohden, Tokyo, Japan).

### Serum and tissues biochemical analysis

Serum triglyceride, total cholesterol, and free fatty acid concentrations were measured by using commercial kits (Wako, Osaka, Japan). The triglycerides and total cholesterol of tissues such as MesWAT and liver were extracted by using a chloroform–methanol solution (2:1, vol:vol) according to the Bligh and Dyer method^65^. Briefly, the chloroform–methanol solution was added to the homogenized liver tissues, which then were vortexed and centrifuged; the lower phase was collected and evaporated at room temperature under a fume hood. The resulting semi-dried pellets were dissolved in 1% Triton X-100 (Nacalai Tesque, Kyoto, Japan). The triglyceride and total cholesterol contents of tissues were analysed by using the same enzymatic kits as those for the serum analyses. The serum concentrations of insulin, leptin, and adiponectin were determined by using ELISA kits (insulin and leptin: Morinaga, Yokohama, Japan; adiponectin: Otsuka Pharmaceutical, Tokyo, Japan). Serum concentrations of TNF-α, IL-6, and MCP-1 were determined by using Quantikine ELISA kits (R&D Systems, Minneapolis, MN, USA).

### Histology and immunohistochemical analysis

Samples of adipose tissue, pancreas, and liver were fixed in 4% paraformaldehyde in phosphate buffer solution (Wako) for 24 h at room temperature, embedded in paraffin, sectioned at 4 μm, and stained with haematoxylin and eosin. For immunohistochemistry, paraffin-embedded sections were stained with monoclonal anti-insulin (Histofine; Nichirei, Tokyo, Japan), anti-Ki67 (dilution, 1:1000; Abcam, Cambridge, MA, USA), and anti-F4/80 (AbD Serotec, Oxford, UK) antibodies. Images were acquired by using an FSX100 system (Olympus, Tokyo, Japan), and the F4/80-positive area was quantified by using cellSens Dimension software (version 1.6, Olympus). Histologic images were analysed to calculate the sizes of adipocytes and of the insulin- and Ki67-positive areas by using Image J software (National Institutes of Health, Bethesda, MD, USA). For the analyses, 5 random images were captured from each sample.

### RNA isolation and quantitative RT-PCR analysis

Total RNA was isolated from tissue samples by using TRIzol Reagent (Invitrogen, Carlsbad, CA, USA). First-strand cDNA was synthesized from total RNA by using a commercially available kit (QuantiTect Reverse Transcription kit; Qiagen, Hamburg, Germany). Quantitative real-time PCR analysis was performed by using the SYBR Premix Ex Taq (Takara, Shiga, Japan), a LightCycler 480 System II (Roche Applied Science, Indianapolis, IN, USA), and gene-specific primers (Supplementary Table S3). Gene copy numbers were calculated by using a standard curve generated from serially diluted plasmid DNA and were normalized against the mRNA level of the ribosomal protein 36B4.

### Western blot analysis

Tissues were lysed in NP-40 buffer supplemented with protease and phosphatase inhibitor cocktails (catalogue nos. 04080-11 and 07574-61; Nacalai Tesque, Kyoto, Japan). Total protein concentrations were determined by using the Pierce 660 nm Protein Assay Reagent (Thermo Fisher Scientific, Rockford, MA, USA). Proteins were separated by electrophoresis through a 4–15% sodium dodecyl sulphate–polyacrylamide gel (Bio-Rad, Hercules, CA, USA) and were transferred to a polyvinylidene difluoride membrane (Millipore, Billerica, MA, USA). The membrane was incubated in blocking reagent (Toyobo, Osaka, Japan) at room temperature for 20 min and then was incubated at 4 °C overnight with the appropriate primary antibody diluted in Can Get Signal Solution 1 (Toyobo). All primary antibodies were from Cell Signaling Technology (Danvers, MA, USA): anti-phospho-Akt (Ser^473^) (catalogue no. 9271), anti-Akt (catalogue no. 9272), anti-ACC (catalogue no. 3662), anti-FASN (catalogue no. 3180), and anti-GAPDH (catalogue no. 2118). An image of the membrane was obtained by using a LAS-4000 mini luminescent image analyser (GE Healthcare UK, Little Chalfont, England, UK), and band intensities were quantitated by using Multi Gauge software (version 3.11, GE Healthcare UK).

### Glucose and insulin tolerance tests

Mice were fasted overnight (~16 h), and GTT was performed by using 1 g (E-CNP Tg) or 0.625 g (OBEC) of glucose per kilogram body weight, administered by intraperitoneal injection. Blood glucose levels were measured at the indicated time points before and after glucose challenge. For ITT, mice were fasted for 4 h prior to intraperitoneal injection of insulin (0.75 U/kg body weight; Eli Lilly, Indianapolis, IN, USA). Blood samples were drawn from the tail vein for measurements of blood glucose by using a glucometer (Sanwa Kagaku Kenkyusho, Nagoya, Japan).

### Statistical analysis

All values are expressed as means ± SEM. Data was analysed by using SPSS software (version 12.0 for Windows; IBM, Armonk, NY, USA). Statistical significance was evaluated by using Student’s *t*-test and one-way ANOVA with *post hoc* Tukey–Kramer testing. Differences were considered significant when *P* values were less than 0.05.

## Electronic supplementary material

Supplementary information
