# Supplementary material for: Overexpression of C-type Natriuretic Peptide in Endothelial Cells Protects against Insulin Resistance and Inflammation during Diet-induced Obesity
Source: Sci Rep. 2017 Aug 29;7:9807. doi: 10.1038/s41598-017-10240-1 (PMC5574992; doi:10.1038/s41598-017-10240-1)
Supplement: Supplementary file 1 — Supplementary information [file 41598_2017_10240_MOESM1_ESM.pdf]

SUPPLEMENTARY INFORMATION

Overexpression of C-type Natriuretic Peptide in Endothelial Cells Protects against Insulin Resistance and Inflammation during Diet induced Obesity

Cho-Rong Bae[1], Jun Hino[1], Hiroshi Hosoda[2], Yuji Arai[3], Cheol Son[4,5], Hisashi Makino[4], Takeshi Tokudome[1], Tsutomu Tomita[4,6], Toru Kimura[1], Takashi Nojiri[1], Kiminori Hosoda[4], Mikiya Miyazato[1] & Kenji Kangawa[1]

Departments of [1]Biochemistry, [2]Regenerative Medicine and Tissue Engineering, and [3]Bioscience and Genetics, National Cerebral and Cardiovascular Center Research Institute, 5-7-1, Fujishirodai, Suita-city, Osaka 565-8565, Japan; [4]Division of Endocrinology and Metabolism, [5]Omics Research Center, and [6]Biobank, National Cerebral and Cardiovascular Center, Suita-city, Osaka, 565-8565, Japan

SUPPLEMENTARY FIGURES and FIGURE LEGENDS

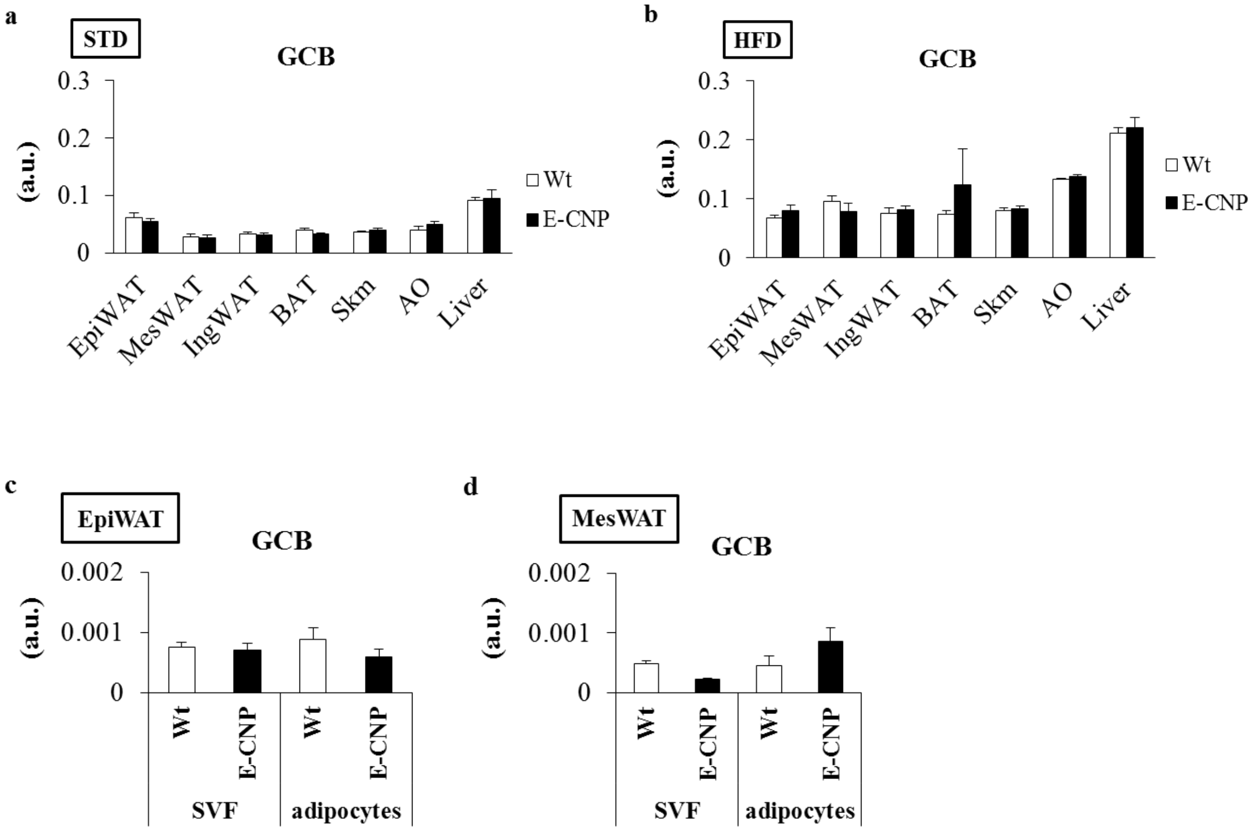

Supplementary Figure S1. (a and b) Guanylyl cyclase B (*GCB*) mRNA levels in tissues from Wt and E-CNP Tg mice (age, 20 weeks) fed (a) STD and (b) HFD were measured by using qPCR analysis. (c and d) *GCB* mRNA levels in the stromal vascular fraction (SVF) and mature adipocytes of (c) EpiWAT and (d) MesWAT from Wt and E-CNP Tg HFD-fed mice. a.u., arbitrary units (copy number of target gene / copy number of reference gene [ribosomal protein 36B4]). a.u., arbitrary units. n = 10 (a and b); n = 4 (c and d). AO, aorta; BAT, brown adipose tissue; EpiWAT, epididymal white adipose tissue; IngWAT, inguinal white adipose tissue; MesWAT, mesenteric white adipose tissue; Skm, skeletal muscle.

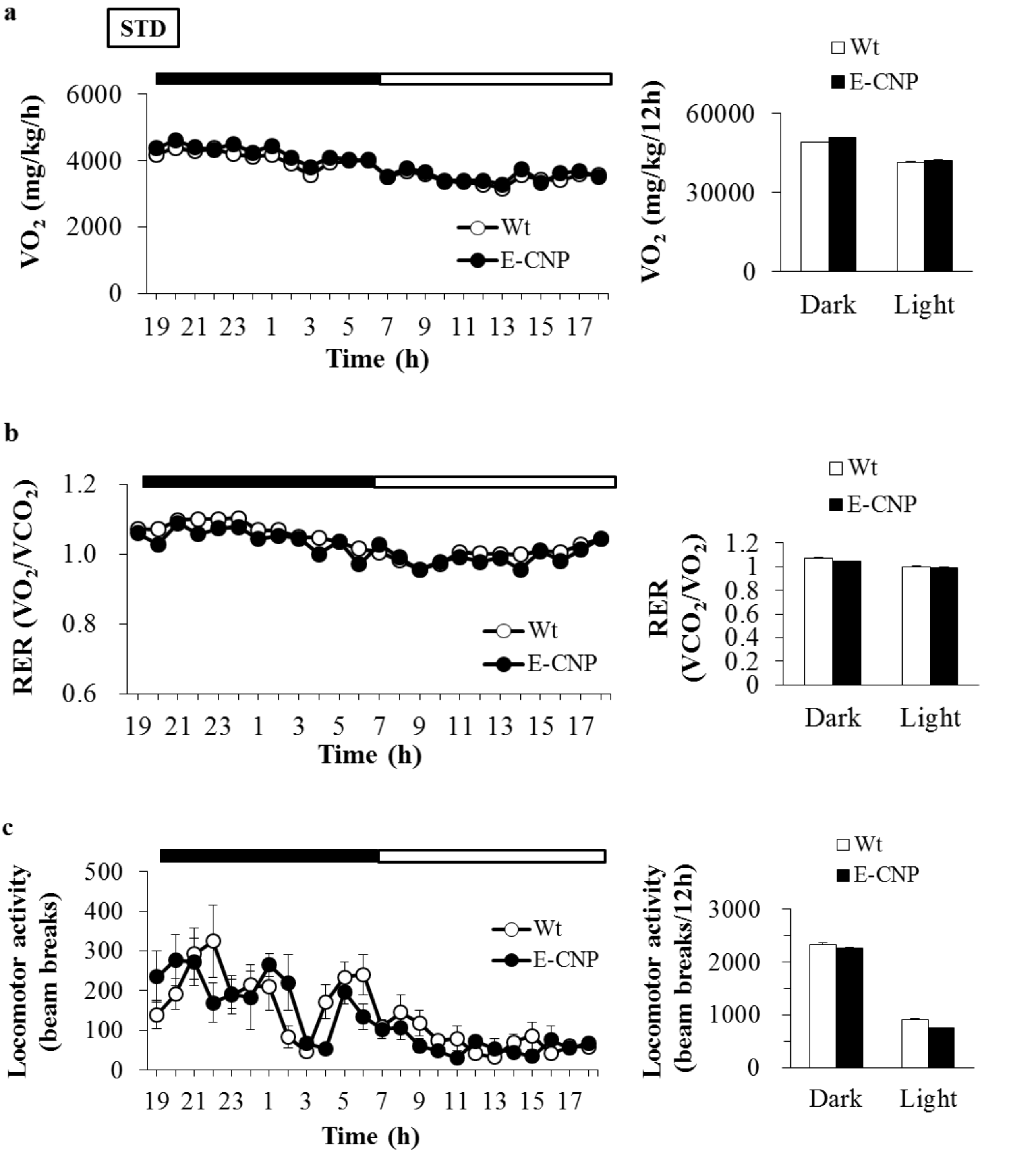

Supplementary Figure S2. Energy metabolism of STD-fed Wt and E-CNP Tg mice. (a) Oxygen consumption (VO_2). (b) Respiratory exchange ratio (RER). (c) Locomotor activity. n = 10 (a–c).

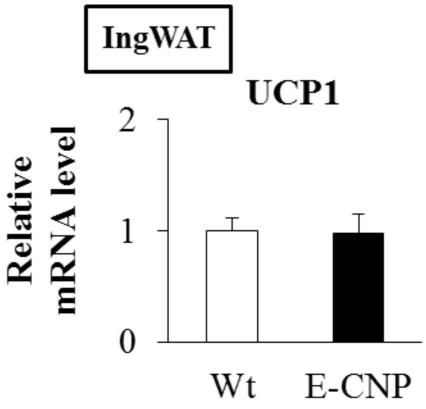

Supplementary Figure S3. qPCR expression analysis of the thermogenesis-related gene *UCP1* in IngWAT. *n* = 10.

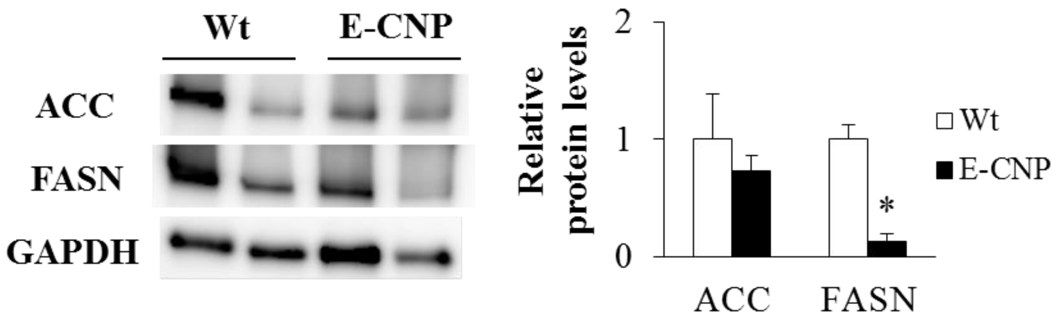

Supplementary Figure S4. Western blot analysis of ACC and FASN levels (lipogenesis-related proteins) in MesWAT extracts. Western blot results were quantified for each lane using program Multi-Gauge. $n = 5$. *, $P < 0.05$.

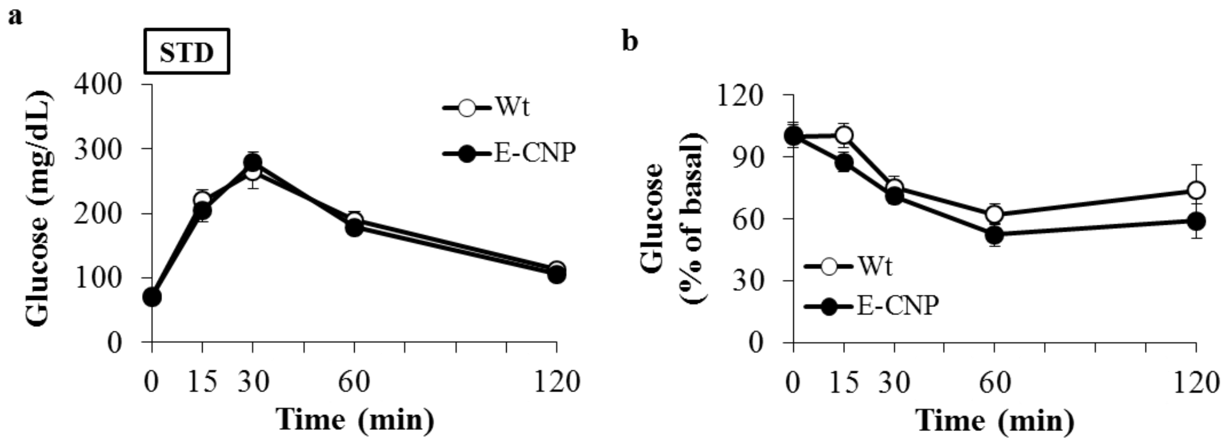

Supplementary Figure S5. Results of (a) glucose tolerance test and (b) insulin tolerance test in 20-week-old Wt and E-CNP Tg mice fed standard diet (STD). *n* = 10 (a and b).

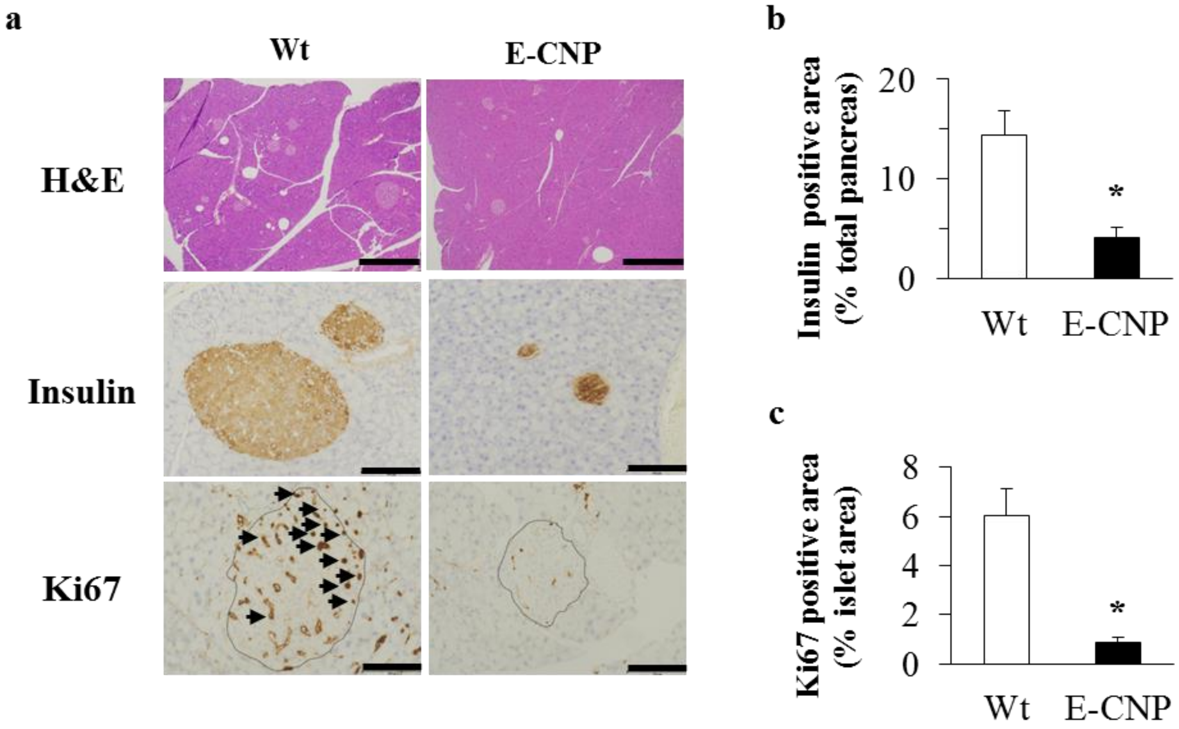

Supplementary Figure S6. (a) Histology (haematoxylin and eosin [H&E] stain) and immunohistochemical quantification of the (b) insulin and (c) Ki67 positive areas in pancreatic tissue from Wt and E-CNP Tg HFD-fed mice. Scale bars: H&E, 500 μm; insulin and Ki67, 100 μm. $n = 9$ (a–c); *, $P < 0.05$.

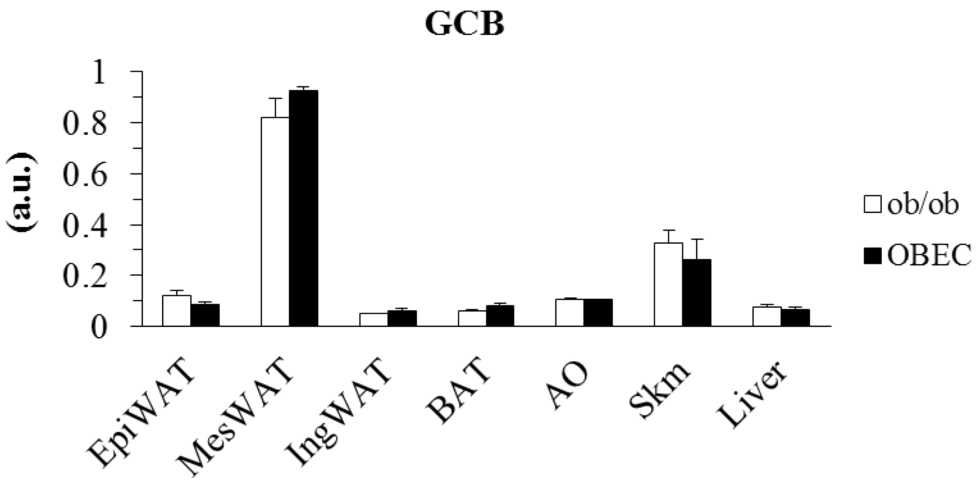

Supplementary Figure S7. *GCB* mRNA levels in tissues (EpiWAT, MesWAT, IngWAT, BAT, AO, Skm, and liver) of ob/ob and OBEC mice (age, 10 weeks) were measured by using qPCR analysis. OBEC, ob/ob・ob/ E-CNP Tg mice. a.u., arbitrary units (copy number of gene of interest / copy number of reference gene [ribosomal protein 36B4]). n = 10.

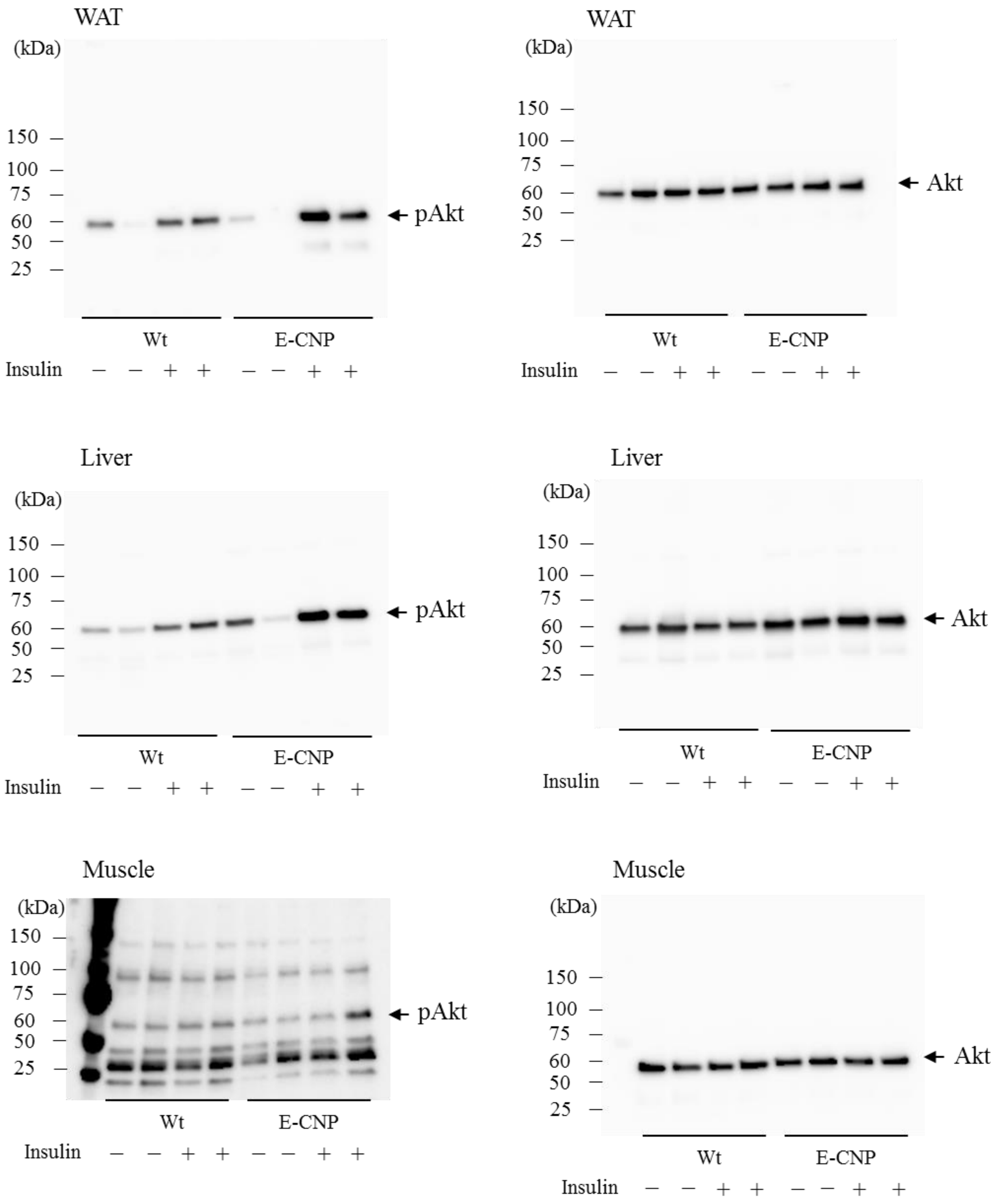

Supplementary Figure S8. The full-length blots are display of cropped blots from the main Figure 5d.

SUPPLEMENTARY TABLES and TABLE LEGENDS

Supplementary Table S1. Serum parameters in HFD-fed Wt and E-CNP Tg mice

	Wt	E-CNP
Triglycerides (mg/dL)	43.93 ± 4.2	30.4 ± 4.3*
Total cholesterol (mg/dL)	247.25 ±7.8	212.9 ± 13.7*
Free fatty acids (mEq/L)	0.66 ± 0.02	0.65 ± 0.03
Insulin (ng/mL)	1.8 ± 0.2	1.1 ± 0.2*
Leptin (ng/mL)	35.5 ± 0.6	31.7 ± 1.4*
Adiponectin (ng/mL)	1.7 ± 0.05	2.1 ± 0.2*

Values are shown as means ± SEM (n = 10).

*, Value differs significantly ($P < 0.05$) between Wt and E-CNP Tg mice.

Supplementary Table S2. Blood pressure in Wt and E-CNP Tg mice

	Blood pressure (mm Hg)			Heart rate (bpm)
	Systolic	Mean	Diastolic	
Wt	112.2 ± 3.1	76.2 ± 1.3	58.3 ± 2.3	678 ± 24
E-CNP	109.7 ± 2.1	80.5 ± 2.1	66.0 ± 2.8	686 ± 21

Values are shown as means ± SEM (n = 5).

Supplementary Table S3. Primers used for real-time PCR analysis of gene expression

Gene	Forward Primer (5′ to 3′)	Reverse Primer (3′ to 5′)
ACC	CCCATCCAAACAGAGGGAAC	CTGACAAGGTGGCGTGAAG
ChREBP	GGCCTGGCTGGAACAGTA	CGAAGGGAATTCAGGACAGT
Cidea	TGCTCTTCTGTATCGCCCAGT	GCCGTGTTAAGGAATCTGCTG
CNP	ACCGAAGGTCCCGAGAACCCC	GACTTGGTGTCCACACGCAGGTCC
CPT1	TGCACTACGGAGTCCTGCAA	GGACAACCTCCATGGCTCAG
F4/80	CTTTGGCTATGGGCTTCCAGTC	GCAAGGAGGGCAGAGTTGATCGTG
FASN	GCTGCTGTTGGAAGTCAGC	AGTGTTCGTTCCTCGGAGTG
GCB	GTCGCTGCGGGGATCCAGTTACG	ATGTTGGGAGGGTCTATGCAGGC
HSL	GCGCTGGAGGAGTGTTTTT	CCGCTCTCCAGTTGAACC
IL-6	CCAGTTGCCTTCTTGGGACTGATG	GTAATTAAGCCTCCGACTTGTGTGAA
MCP-1	GCAGGTGTCCCAAAGAAGCTGTAG	CAGAAGTGCTTGAGGTGGTTGTGG
PGC1α	CCCTGCCATTGTTAAGACC	TGCTGCTGTTCCTGTTTTC
PPARα	CTGAGACCCTCGGGGAAC	AAACGTCAGTTCACAGGGAAG
PPARγ	AGGCCGAGAAGGAGAAGCTGTTG	TGGCCACCTCTTTGCTCTGCTC
PRDM16	TGGCCTTCATCACCTCTCTGAA	TTTCTGATCCACGGCTCCTGTGA
SREBP1c	TTCCTCAGACTGTAGGCAAATCT	AGCCTCAGTTTACCCACTCCT
TNF-α	TGGCCCAGACCCTCACACTCAGATC	GCCTTGTCCCTTGAAGAGAACCTGG
UCP1	ACTGCCACACCTCCAGTCATT	CTTTGCCTCACTCAGGATTGG
36B4	TCATTGTGGGAGCAGACAATGTGG	AGGTCCTCCTTGGTGAACACAAAG

ACC, acetyl CoA carboxylase; ChREBP, carbohydrate responsive element–binding protein; Cidea, cell death-inducing DNA fragmentation factor α-like effector A; CNP, C-type natriuretic peptide; CPT1, carnitine palmitoyltransferase 1; FASN, fatty acid synthase; GCB, guanylyl cyclase B; HSL, hormone-sensitive lipase; IL-6, interleukin-6; MCP-1, monocyte chemoattractant protein-1; PGC1α, peroxisome proliferator–activated receptor γ coactivator 1α; PPARα, peroxisome proliferator–activated receptor α; PPARγ, peroxisome proliferator–activated receptor γ; PRDM16, positive regulatory domain-containing protein 16; SREBP1c, sterol regulatory element–binding protein 1c; TNF-α, tumor necrosis factor-α; UCP1, uncoupling protein 1; 36B4, acidic ribosomal phosphoprotein P0.

SUPPLEMENTARY METHOD

Measurement of blood pressure and heart rate

Blood pressure and heart rate were measured in conscious animals using the tail-cuff method (Softron, BP-98A, Tokyo, Japan).
